# The State of Lunacy in Scotland

**Published:** 1860-07-01

**Authors:** 


### Art. VI.—THE STATE OF LUNACY IN SCOTLAND.

The Second Annual Report of the Scotch Commissioners in Lunacy, now before the public, contains much of interest, as well general as special.

The Commissioners state that, notwithstanding the difficulties which have impeded the effective carrying out of the provisions of the lunacy statute, the treatment of the insane in Scotland "has already undergone a manifest improvement." The difficulties referred to chiefly arose from imperfections in the phraseology and drawing-up of the statute, as we pretty fully set forth in our analysis of the First Annual Report of the Commissioners.* We then remarked, that "never was an Act more ingeniously worded for rendering nugatory the presumed intentions of its framers; never was a Board created to carry out the intentions of an Act, more impressed with the spirit, yet more perplexed with the letter of the law, than the General Board of Commissioners in Lunacy for Scotland." It is consoling, therefore, to find that the Commissioners, notwithstanding the obstacles which have beset them in the performance of their onerous and delicate duties, are able to report a material improvement in the condition of the insane under their observation.

The Commissioners express the fear that there is a steady and serious increase of lunacy in Scotland. On the 1st of January, 1858, the pauper lunatics amounted to 4737; on the 1st of January, 1859, to 4890. "We have no means," the Commissioners state, "of obtaining reliable returns of the numbers of the private insane, with the exception of those placed in asylums, but there is good reason to fear that they are increasing in a similar ratio."—(p. ii.) We shall recur presently to this question of the increase of insanity.

The number of insane under care in Scotland, on the 1st of January, 1859, was 7878—3829 being males, and 4049 females. The distribution of these patients, the manner in which they are supported, and their increase or decrease in number, since the previous returns, in public and private asylums, workhouses, and as single patients, were as follows:—

*Public and District Asylums.*—Number of patients, 2496; males, 1271, females, 1225; increase, 116: supported by private funds, 809, by parish rates, 1687.

*Private Asylums.*—Number of patients, 821; males, 351, females, 470; increase, 76: supported by private funds, 200, by parish rates, 621.

*Poor-houses.*—Number of patients, 797; males, 328, females, 469; decrease, 42. All the patients except two, were maintained by their parishes.

"The decrease in the number of patients in poor-houses is due to the withdrawal from the roll of pauper lunatics of a considerable number of demented and imbecile persons placed in the ordinary wards of these establishments; but who, though formerly reported as fatuous to the Board of Supervision, are now certified by the parochial surgeon as not coming within the provisions of the Lunacy Act." (p. ii.)

*Single Patients.*—The number of pauper lunatics placed as single patients amounted to 1877—838 being males, and 1039 females. "Of these, 688 men and 794 women were living with

* See vol. xii. of this Journal, p. 429.

relatives; 133 men and 197 women were placed with strangers; and 17 men and 48 women were living alone." (p. 5.)

Only 27 private single patients—12 men and 15 women—were reported to the Commissioners in obedience to the requirements of the Act. The Board were, however, through the reports of the Visiting Commissioners, cognizant of the existence of 1887 single patients—1041 men and 846 women; but the majority of these patients were living under the care of relations, and, consequently, did not fall under the provisions of the Act. Large as is the number of private single patients known to the Commissioners, they think that it falls considerably short of the number actually existing.

From these figures, then, it would appear that of the 7878 known lunatics in Scotland, 2898 are supported by private funds and 4980 by parochial rates. Further, it would seem that a preference is given by the friends of private patients to public asylums, 809 patients of this class being placed in these institutions and only 200 in licensed houses. This, the Commissioners think, "affords a strong argument in favour of providing accommodation of a superior kind in connexion with the district asylums." (p. iii.)

It is reported, however, in addition, that—

"A very large proportion of the non-parochial patients who are in private houses belong to families so little removed above pauperism, that many of them are detained at home entirely from the inability of friends to pay for their maintenance in asylums. This is a fact of very grave import, and should be constantly borne in mind in all arrangements for providing a national system of asylum accommodation." (p. iii.)

The Commissioners now recur to the question of the increase of insanity, and remark:—

"The experience of all countries has shown, that the numbers of the insane increase so rapidly, that the accommodation provided, however sufficient it may at first have appeared, has in a short time been found inadequate. In France, for instance, the numbers of the insane in public and private asylums amounted, on 1st January, 1835, to 10,539; whereas, on 1st January, 1854, they had increased to 24,524. In England and Wales, the number of pauper lunatics amounted, in August, 1843, to 16,764; of whom 3525 were in county asylums, 2298 in licensed houses, and 4063 in workhouses. On 1st January, 1859, the number of pauper lunatics had increased to 30,318; of whom 14,481 were placed in county or borough asylums, 2076 in registered hospitals and licensed houses, and 7963 in workhouses. It thus appears that in sixteen years the number of pauper lunatics in England and Wales had nearly doubled, and that in 1859 nearly as many were in public and private asylums as were on the roll in 1843. In Scotland we find similar results. According to the returns of the Board of Supervision, the number of insane poor *relieved* during the year ended 14th May, 1847, amounted to 2945, and to 5564 for the year ended 14th May, 1858; thus showing an increase of 2619 in eleven years. These numbers refer to the pauper lunatics *relieved* during the year; but supposing that the moderate deduction of ten per cent. be made to determine the numbers on any stated day, we shall have 2650 as the actual number of insane poor in Scotland on 14th May, 1847. Reference to the preceding table will show, that on 1st January, 1859, there were 2308 pauper lunatics in public and private asylums, and 795 in the lunatic wards of poor-houses. That is, there were in lunatic establishments, in 1859, no less than 3103 pauper patients, or 453 more than the total number of the insane poor in 1847. From the investigations undertaken with the view of determining the amount of accommodation that should be provided in district asylums, we arrived at the conclusion that provision would be required for 4353 pauper lunatics; and, on mature consideration, we are not inclined to consider this estimate as excessive. On the contrary, were we to draw our conclusions from past experience, we should have only too great reason to fear that it would soon prove insufficient. The estimate, it may be well to point out, is founded on the supposition that all pauper lunatics are to be accommodated in district asylums, or asylums recognised as efficient substitutes, and presupposes the extinction of all licensed houses and lunatic wards of poor-houses. On this supposition, additional accommodation would be required for 2666 patients, as this number, with the 1687 in public asylums on 1st January, 1859, makes up the estimate of 4353. But, during 1859, additional accommodation for about 400 patients has been provided by the opening of the new asylum of Montrose, and the enlargement of the Southern Counties Asylum at Dumfries; so that the further accommodation now absolutely required, supposing the old asylum at Montrose to remain in permanent operation, is only for 2266 patients. Of these 2266, however, 1416 are already in licensed houses and lunatic wards of poor-houses, so that the actual deficiency of any kind of accommodation is only for 850." (pp. iii., iv.)

Subsequent remarks of the Commissioners show that they are aware that the foregoing figures cannot be looked upon as indicating positively an *absolute* increase in the number of insane in Scotland. The increase spoken of actually refers, on the one hand, to the augmentation of *pauperism* from insanity, on the other, to the increased number of *known* lunatics. There does not seem to be in Scotland, any more than in England, trustworthy data from which a correct notion of the status of lunacy among the population at large may be gathered; and the rate of increase of provision for the insane in both countries has never been sufficient to exhaust the substratum of chronic cases of insanity for which provision is required. The Scotch statistics of lunacy confirm the opinion, that we have oftentimes expressed, of the great importance of adopting some means of determining the actual amount of lunacy in the country at large, and of the chief causes which foster it among the impoverished classes, if we would effectively deal with the great public questions of the care of lunatics and prevention of lunacy.

The Commissioners reiterate their objections to the residence of pauper lunatics in licensed houses and poorhouses, and they revert to the imperfect and perplexing definition of lunacy in the Statute—a subject fully discussed by them in their first report, and which we examined at length in the analysis of that report already referred to. The question, however, is one which cannot be kept too prominently before the public, and we do not hesitate to quote the following additional remarks:—

"Many persons who are totally unfit, from mental aberration or mental deficiency, to take care of themselves, and who, in a court of law, would not be held responsible for their actions, are not regarded by some medical men as coming within its meaning. According to these practitioners, insanity must be of a dangerous character to come within the statutory definition; but it is often extremely difficult to determine what patients should be considered as so affected; for the question of danger is a relative one, and must be determined as much by the circumstances in which the lunatic is placed, as by his peculiar mental condition. Accordingly, many patients who, when in asylums, are very properly regarded as not dangerous, from being under effective surveillance and control, become dangerous as soon as they are discharged, and are allowed to follow the bent of their diseased imaginations. It is in regard to this class of the insane that much trouble is frequently experienced by the superintendents of asylums; for it is often no easy matter to convince the relatives of such patients that the improvement observable in their condition is due, not so much to any essential change in the character of their malady, as to the continuous discipline to which they are subjected in the asylum. When, therefore, a medical practitioner grants a certificate that an insane person is 'not a lunatic in the meaning of the Act,' it is obvious that he thereby incurs a double responsibility, as he must be held to give an opinion, first, in regard to the mental state of the patient, and secondly, as to the appropriate nature of the circumstances in which he is placed. It is, at the same time, evident, that a certificate to the effect that any one is 'not a lunatic in the meaning of the Act,' does not necessarily imply that the person is of sane mind. Indeed, it is frequently expressly understood that the certificate is not intended to convey this meaning, but is granted merely as an expression of opinion that the patient is not likely to commit an act dangerous to himself or others." (p. v.)

The unfortunate operation of the definition, acted upon in the fashion just recounted, has been already illustrated in one respect, in the paragraph containing the summary of the number of lunatics in poor-houses.

The distribution of pauper lunatics in different districts is illustrated by a series of elaborate tables, the data contained in which show that easy access to asylums greatly influences the distribution of the cases. The Commissioners would set it down as an axiom that "the number of patients sent to asylums diminishes in a ratio corresponding to the distance, and that the number of those which remain at home increases in a similar degree."—(p. ix.) Thus in the Forfarshire, Edinburgh, Glasgow, and Renfrewshire districts, which are those most fully provided with accommodation, 83 per cent. of the pauper lunatics are placed in asylums or the lunatic wards of poor-houses; but in the destitute districts of Inverness and Argyle, only 34 per cent. of the pauper lunatics are so disposed of. A further examination of the figures in the tables clearly shows that these differences in the number of pauper lunatics placed under care in the different districts, depends entirely upon the facilities afforded, by easy communication, to placing patients in asylums, and the Commissioners conclude that "small asylums in convenient situations will more satisfactorily meet the wants of the country than large central establishments, which must necessarily be remote from considerable portions of the extensive districts which they are designed to accommodate." (p. x.)

Another question examined in the first report, the difficulty of determining the proportion of patients who should be placed in asylums, and of those who should be left at home, is again touched upon by the Commissioners. They hold that a much larger proportion of lunatics may be properly left at home in rural than urban districts. They remark:—

"This result chiefly depends on the greater difficulty of affording insane persons exercise and recreation, and in otherwise providing for their proper care and treatment, in the town than in the country; and herein lies the reason why, in reality, urban parishes have comparatively so few pauper lunatics placed as single patients. The city of Glasgow parish, for example, has only 23 out of 293 pauper lunatics so disposed of; the Barony Parish, 22 out of 151; Edinburgh City Parish, 8 out of 196; and St. Cuthbert's Parish, 22 out of 171. And if we extend our inquiries to England, we find that of the 4661 pauper lunatics chargeable to the parishes of the metropolis on the 1st January, 1859, only 129 were left in charge of relatives, or were boarded with strangers." (pp. x., xi.)

The Commissioners then add:—

"In all probability, a much larger proportion of the insane poor of these urban parishes would, under different circumstances, have been left at home; and if this be the case, it follows, that in populous districts many patients are placed in asylums, not so much from a regard to their comfort or welfare, as to the convenience of those who have to provide for their maintenance. These patients demand no special curative treatment, but simply such medical and general care as is required by their decayed mental and physical condition. On the other hand, however, there can be no doubt that in rural districts many patients are left at home in pitiable wretchedness, whose condition is capable of great improvement by removal. There is thus a considerable number of lunatics, comprehending, in the first place, those who in cities are sent to asylums, but who, if in rural districts, might with propriety have been left at home; and, in the second place, those in rural districts who are beyond the hope of cure, but whose neglected and miserable condition demands that they should be placed under care, for whom we are of opinion that some kind of modified asylum accommodation should be provided. We strongly object to lunatic wards in poorhouses being used for this purpose, chiefly on the ground that the primary object of poorhouses is to afford a test for poverty, and to provide for the poor in the most economical manner. The fundamental principle on which these establishments are conducted is thus antagonistic to that which ought to regulate the treatment of lunatics, and which, briefly stated, is the provision of every comfort which can reasonably be demanded to lighten the burden of perhaps the greatest calamity which can afflict humanity." (p. xi.)

The progress of the District Lunacy Boards, in providing accommodation for the insane poor, it would appear, is not altogether satisfactory. The Commissioners record the proceedings of the different boards within the period over which the report extends.

The expenditure for pauper lunatics is next discussed, and sundry items of highly interesting information are to be found under this head. It is reported that the highest average rate of maintenance occurs in the county of Nairn, where it amounts to 22*l.* 17*s.* 2*d.*, while the lowest is found in Shetland, the sum being there only 10*l.* 2*s.* 9*d.* No useful comparison between the condition of patients in different counties can, however, be founded upon a mere statement of the money expenditure. The total expenditure by parochial boards on account of pauper lunatics, for the year 1858, was upwards of 81,000*l.* The average expenditure for each lunatic was 16*l.* 5*s.* 4¼*d.*, being at the rate of 27*l.* 19*s.* 1½*d.* for each 1000 of the population, according to the census of 1851. The average cost of maintenance for each pauper-patient in asylums was 21*l.* 18*s.* 2¼*d.*; in poor-houses, 13*l.* 13*s.* 10*d.*; and in private houses, 7*l.* 12*s.* 10*d.*

But the rough averages last quoted are not, it would seem, to be received as presenting an accurate idea of the comparative cost of lunatics in asylums and poor-houses. This is the first attempt that has been made to estimate the cost of pauper lunacy in Scotland; but the parochial accounts not having been kept with a view of distinguishing between the expenditure for sane and insane persons, the results presented by the Commissioners are not to be looked upon as free from doubt. Taking, however, the returns of expenditure as they stand, some curious and unexpected results are obtained from them, having a most important bearing upon the debated question of the economy of transferring lunatics from poor-houses to asylums. The Commissioners institute the following comparative examination of expenditure for pauper lunatics in several parishes :—

"There are certain parishes, for example, which send all their lunatics to asylums, with the exception of those exempted as single patients. There are others which place them preferentially in the lunatic wards of poorhouses; and others, again, which divide them between asylums and poorhouses,—sending to the former the recent and unmanageable cases, and placing in the latter the chronic and more tractable. To the first class belong the parishes of Dumfries, Dundee, Elgin, Liff and Benvie, and Montrose. To the second, the parishes of the Abbey and Burgh, Paisley, and those of the Barony, Falkirk, and Greenock; and to the third, the parishes of Aberdeen, Edinburgh, Glasgow, Old Machar, St. Cuthbert's, and South Leith. In this list, we have comprehended only those parishes which are similarly placed as to the facility of obtaining accommodation, whatever its nature may be; and have purposely excluded such parishes as Inverness and Perth, where distance or other circumstances would have introduced disturbing elements. Inverness, for instance, is altogether dependent for asylum accommodation on remote establishments; and Perth, with a public asylum close at hand, sends 30 patients to distant licensed houses. It is therefore obvious that they are placed in exceptional positions." (p. xxvii.)

Now the general average cost per head of the pauper lunatics of the first class of parishes amounts to 15*l.* 7*s.* 2*d.* (maximum average, 22*l.* 16*s.* 1*d.*, minimum, 11*l.* 3*s.* 6*d.*); of the second class of parishes, 19*l.* 4*s.* 11¼*d.* (maximum average, 20*l.* 13*s.* 4*d.* minimum, 18*l.* 0*s.* 8½*d.*); of the third class of parishes, 17*l.* 1*s.* 5¾*d.* (maximum average, 18*l.* 16*s.* 3¼*d.*, minimum, 15*l.* 11*s.* 9*d.*)

The Commissioners remark that,—

"These results are extremely important as indicating that asylum treatment is really more economical than poorhouse treatment. They show that parishes which take the entire charge of their pauper lunatics, and treat those requiring segregation entirely in the lunatic wards of poorhouses, maintain the whole at an average rate of 19*l.* 4*s.* 11¼*d.* per head; that those parishes which place only the more manageable of their patients requiring segregation in poorhouses, and send the rest to asylums, maintain the whole at an average rate of 17*l.* 1*s.* 5¼*d.*; and lastly, that those parishes which trust entirely to asylums for the care and treatment of such of their lunatics as require to be placed in establishments, maintain the whole at an average rate of 15*l.* 7*s.* 2*d.*

"It is occasionally difficult to account for the differences in the returns made by parishes which, to all appearance, are in precisely similar circumstances. Thus, we are quite unable to adduce any satisfactory reason for the great difference which exists between the cost of the pauper lunatics of the parish of Dundee and that of Liff and Benvie. These parishes are contiguous, and equally conveniently situated in regard to the Dundee Asylum, to which both have right of admission for their pauper lunatics at privileged rates; yet there is a difference of not less than 6*l.* in the average cost of their pauper lunatics, that for Liff and Benvie being 22*l.* 16*s.* 1*d.*—a sum which is 2*l.* 0*s.* 1*d.* above the rate of maintenance charged by the asylum. Accordingly, in making the foregoing comparisons, we wish it to be distinctly understood that we regard the results as by no means free from doubt. In the first place, as already stated, the parochial accounts are generally so kept that no distinction is made in the expenditure for sane and insane paupers. In a great measure, then, the returns sent us must be founded on probable estimates; which may be near the truth, but, on the other hand, may be very inaccurate. In the second place, the rate of maintenance, in the case of some poorhouses, comprises a charge for rent, which is an item not included in asylum rates, or only to a partial extent in the shape of interest on debt. Nevertheless, after making due allowance for all possible sources of error, it appears tolerably certain that not only does no economical advantage accrue to a parish by converting part of its poorhouse into lunatic wards, but that the practice entails a positive loss, which becomes the greater, the more exclusively such wards are had recourse to. And it should likewise be borne in mind, that this result is not compensated by any gain to the patients. On the contrary, they are deprived of many comforts and sources of occupation and amusement which they enjoy in asylums." (p. xxviii.)

Much misconception existing as to the probable future expenditure for pauper lunatics, the Commissioners examine the question, and come to the conclusion that the cost, after the establishment of district asylums, will probably not exceed the present amount.

The Commissioners have adopted a system of statistics which they hope will ultimately give correcter results than those usually obtained, of the influence of treatment upon, and the increase of lunacy.* The method they have put into operation is—

"To determine the number of patients admitted each year into all

* The Commissioners correct an error in the report of the Irish Lunacy Inspectors for 1859, which it is requisite to note, as we quoted the error in our review of that report. The Irish Inspectors "contrasted the absolute cures in Irish asylums, estimated at 48·71 per cent. on the admissions, with those in the Scotch asylums, estimated at 36·99 per cent. The estimate, however, as regards the latter establishments, is correct only for 1858, in which year the transfers of incurable patients were very numerous, and of course reduced in a corresponding degree the proportion of recoveries in the asylums receiving them." (p. xxxii.)

asylums, distinguishing between recent and chronic cases, that is, between cases which for the first time were admitted into asylums, and those which had previously been under treatment in such establishments. For a few years the admissions of cases regarded as recent will necessarily include a certain number of patients who had been in asylums before the Statute under which we act came into operation, and of whose previous history we have no record. But in the course of time, this source of fallacy will gradually become less, and we shall then be able to draw a tolerably accurate line between recent and chronic cases. . . . . We propose keeping the results of each year apart, so as to be able to determine the number of patients who became insane in each year, and the relative proportions of those who were cured, were discharged unrecovered, or who died in that or in any following year. We shall thus be enabled to follow the history of all the patients who became insane in any given year, or rather, who were then, for the first time, admitted into any asylum in Scotland, through all subsequent changes, until they are finally disposed of by recovery, removal, or death." (p. xxxiii.)

The following tables contain the results thus obtained for 1858 and 1859 :—

Progressive History of Patients admitted for the first time into Asylums in 1858.

| | | Number of Patients admitted and re-admitted during year. | Number of Patients discharged recovered during year. | Number of Patients discharged not recovered during year. | Died during year. | Patients remaining at close of year. |
|---|---|---|---|---|---|---|
| **Results of 1858.** | | | | | | |
| New Patients admitted in 1858 . | 1308 | | | | | |
| Of these there were re-admitted during the course of the same year . . . . . . . . . . | 30 | | | | | |
| | | 1338 | 338 | 68 | 108 | 824 |
| **Results of 1859.** | | | | | | |
| Remainder at 1st January, 1859, of the 1308 new Patients admitted during 1858 . . . . | 824 | | | | | |
| Patients re-admitted during 1859 of the original 1308 cases of 1858 | 74 | | | | | |
| | | 898 | 194 | 28 | 77 | 599 |

* The numbers under the heads of Re-admissions and Discharges refer to the number of individual patients re-admitted and discharged, and do not show how often the same patient may have been admitted and discharged. For instance, of the thirty patients re-admitted in 1858, some of them have been discharged and re-admitted twice, or even thrice; but in the columns of re-admissions and discharges, all discharges and re-admissions of the same patient count only once. This method

Progressive History of Patients admitted for the first time into Asylums in 1859.

| Results of 1859. | | Number of Patients admitted and re-admitted during year. | Number of Patients discharged recovered during year. | Number of Patients discharged not recovered during year. | Died during year. | Patients remaining at close of year. |
|---|---|---|---|---|---|---|
| New Patients admitted into Asylums in 1859 . . . . . . | 1226 | | | | | |
| Of these there were re-admitted during the course of the same year . . . . . . . . . | 29 | | | | | |
| | —— | 1255 | 302 | 76 | 105 | 772 |

TABLE showing the changes which occurred in the numbers and condition of PAUPER LUNATICS registered as SINGLE PATIENTS during the year 1858 :—

| Number of Single Pauper Lunatics on 1st January, 1858. | New cases registered during year. | | Total on Register during year. | Removed from Register during year by | | | | | | Total Removals from Register. |
|---|---|---|---|---|---|---|---|---|---|---|
| | Intimated by Inspectors. | Removed from Asylums. | | Removal to Asylums. | Death. | Recovery. | Removal from Poor-roll. | Removal from Roll of Lunatics. | Cause of removal not intimated.* | |
| 1784 | 390 | 40 | 2214 | 112 | 104 | 46 | 25 | 1 | 49 | 337 |

Although, as we have already stated, the condition of the insane in Scotland has undergone a manifest improvement since the formation of the Lunacy Board, the Commissioners state that that condition is still far from satisfactory; and after giving certain details illustrative of the extent of their visitations in the kingdom and the degree of improvement effected by them, they offer several suggestions of great interest respecting the future provision for lunatics. We shall quote these suggestions in full, the subject they deal with being perhaps the most difficult of the many difficult questions which beset lunacy legislation and management.

has been adopted to make the numbers remaining at the end of the year tally with the numbers withdrawn. Had each discharge or re-admission been counted as a separate case, it is obvious that the numbers at the end of the year would have stood in no relation to the original numbers, and that great confusion would have ensued.

* There is no statutory requirement for inspectors to give intimation of removal from Roll; and in many cases, accordingly, we learn the fact only by the omission of the names in the next annual return. It is probable that death is the chief cause of the removals under this head.

"While thus adverting to the benefits accruing from visitation, we do not conceal from ourselves the difficulty, we may almost say the impossibility, of exercising sufficient surveillance over patients who are scattered over the whole country. That all cases of insanity should be placed in asylums is a proposition which we cannot entertain; the welfare of the patients would not thereby be promoted, while the expense to the country would undoubtedly be greatly increased. But neither are we disposed to consider it a judicious arrangement that so-called harmless or fatuous patients should be congregated together in the lunatic wards of poorhouses. All great aggregations of permanently diseased minds are evils which should as much as possible be avoided, as their tendency is undoubtedly to lower and degrade each constituent member of the mass. Viewed in a certain light, then, asylums may be regarded as necessary evils; but in no view, save in the doubtful one of economy, can the establishment of lunatic wards in poorhouses, in which only chronic or fatuous patients shall be received, be regarded as otherwise than injudicious. These poorhouse wards are simply convenient receptacles for patients affected with chronic insanity or imbecility, in which their physical wants are more or less adequately supplied, but in which little or nothing is attempted, by means calculated to exercise the limited faculties which yet remain to them, to break the weary monotony of prolonged confinement. Many lunatics and imbeciles, though with perverted intelligence or deficient mental powers, have still warm affections, and are capable of deriving enjoyment from social intercourse. Others, again, though wayward and capricious, are much more likely to be manageable in small communities, than where, in large numbers, they are confided to the care of attendants, frequently of an inferior class, who have neither the will nor the capacity to make allowance for their peculiarities.

"We have already alluded to the statutory enactment, that not more than one lunatic shall be placed in any house which is not licensed. As the licence fee amounts to 15*l*. 10*s*. per annum, this provision is an effectual obstacle against the introduction of a more home-like system of accommodation than that at present in use. We are very anxious, therefore, to see some change effected in this respect; and we are of opinion that, were the restriction alluded to removed, an efficient system of domestic accommodation would gradually be developed for such of the insane as were not proper patients for asylums. On this account we should gladly see it enacted, that any number of patients not exceeding four might be received into a private house, without the necessity for a licence, provided the board made previous inquiry into the nature of each case, and granted their sanction according to special forms for the admission of each individual patient. Under some such provision we feel satisfied a system of cottage accommodation would gradually spring up, which would not only furnish more fitting accommodation for chronic patients than the lunatic wards of poorhouses, but would also be calculated to prove a valuable adjunct to asylums. The practical advantages of such a system would be, first, increased comfort to the patients; secondly, greater economy to the parishes; and thirdly, diminished labour of visitation to the Commissioners.

Were we to decide on the first point simply by the wishes of the patients themselves, or by those of their relatives, we could have no hesitation in at once accepting it as proved; but, apart from these considerations, we are satisfied from observation, that cottage accommodation, if placed under efficient supervision, would be found to possess many advantages over poorhouses. These advantages are chiefly the greater amount of liberty accorded to the patients; their more domestic treatment; and their more thoroughly recognised individuality. In regard to the point of economy, we have only to recall the fact, that in poorhouses the annual average cost is 13*l.* 13*s.* 10*d.* for each pauper lunatic; and that for four patients the amount would thus be 54*l.* 15*s.* 4*d.* Now, our returns show that the annual average cost per head of pauper lunatics placed singly is only 7*l.* 12*s.* 10*d.*, or 30*l.* 11*s.* 4*d.* for four. This sum, however, we consider as quite inadequate for the entire maintenance of a patient, and in reality it must generally be regarded only as a subsidy given by the parish to assist in his support. But we are of opinion, that from 12*l.* to 15*l.* a head, where three or four patients are placed together, would prove inducement sufficient to bring forward persons of respectable character to undertake their entire care and support; and, as has been stated, it is on the introduction of this system that we ground our hopes of so restricting the number of patients in asylums as to keep the general expenditure for pauper lunatics within 100,000*l.* per annum. We calculate that about one-fifth of the total number would still be left with relatives at an average rate of 8*l.*, so that any diminution in the expenditure would be the result of the development of the cottage system, and its application to cases at present retained in asylums. By its adoption economy would ensue, not only from the smaller cost of maintenance, but also from the diminished necessity for providing expensive asylums, as we have no doubt that appropriate cottage accommodation would cost materially less. We do not, however, conceal from ourselves the obstacles likely to be encountered in introducing a system such as that proposed; but we are, at the same time, convinced that these would be found by no means insurmountable, and that the result would be most beneficial to the country. We are not, however, desirous for any sudden or sweeping alteration of the present system, but simply for the removal of the legal difficulties which prevent the reception of more than one patient without a license. To the Visiting Commissioners the advantages would also be great. By placing three or four patients together, the number of houses requiring visitation would be greatly lessened; and the labour of inspection would be further diminished were the cottages generally grouped together. If, as we hoped would be the case, they were usually erected in the neighbourhood of asylums, an interchange of patients would naturally and easily take place, whenever any alteration in the character of the mental or bodily condition of the patients rendered it desirable. Indeed, under such circumstances, the cottages might be regarded simply as an out-lying part of the asylum.

In these remarks we refer more especially to pauper lunatics; but we believe there are many private lunatics who might be accommodated in a like manner, greatly to the relief of relatives, and with increase of comfort to the patients. We are aware of the existence of 1887 private patients not in asylums, of whom a very large proportion are in indigent or even in destitute circumstances. In many cases, the relatives of these patients struggle on without applying for parochial relief; and, in many others, application is made only to be refused, and much misery is thus endured. We are aware that the proper mode of affording relief to the poor forms one of the most difficult problems in economic science; and this question, moreover, is one which it does not fall within our province to determine. It is, however, our duty to point out the many evils which a refusal of relief too often entails upon the patient and his relatives. In the first place, the malady is allowed to pass into an incurable form, and the patient is rendered unproductive for life. In the second place, the small means of the family are gradually dissipated in the struggle, and the whole are reduced to the condition of paupers. Finally, the habitual presence of an insane person is apt to induce the disease in others, especially when there happens to be a hereditary tendency. The comfort of the household is destroyed; habits of regularity and industry are broken through; and, not unfrequently, the constant sight of the sufferer engenders a feeling of despair, and induces the habitual resort to intoxicating liquors." (pp. xli.—xiv.)

These suggestions are well worthy of serious consideration. They indicate one mode of providing for lunatics of a certain class, which both medically and economically commends itself to our approbation, and which would appear to be feasible. An additional interest is given to the suggestions of the Scotch Lunacy Commissioners, when they are compared with Dr. Parigot's observations on the Belgian "free air system" of treating lunatics, contained in a paper on the reform of lunatic asylums, to be found in the present number of this Journal.

The impediments cast in the way of the Commissioners, in the performance of their duties, by parish authorities, although descanted upon by them, need but a passing notice from us. Beadledom is the same all the world over. But we are much interested in a comparison which the Commissioners institute between the public asylums of Scotland and England, seeing that the chartered asylums of the former country have been so recently held up by the Chairman of our Lunacy Board, the Earl of Shaftesbury, in his official capacity, for imitation by us. The Scotch Lunacy Commissioners say:—

"During the past year the condition of the public asylums has, on the whole, continued to improve, although, in several respects, it falls considerably below the general standard of English county asylums. But in making this comparison, we must direct attention to the fact, that in one very essential respect the Scotch asylums do not occupy nearly so favourable a position as those of England. In the latter country, the necessary funds are raised by assessment; and an asylum, calculated to afford accommodation for all the patients of the county, and supplied with all the necessary appliances, is at once provided. Should this accommodation be afterwards found to be insufficient, a further assessment is made and additional buildings are erected. In Scotland, on the other hand, the directors of the public asylums possess no compulsory powers of raising funds. The houses have been built with money derived from legacies, charitable donations, and subscriptions; and their extension chiefly provided for by the payments made for patients. The cost of the original building, and its subsequent extension, have thus both been defrayed from uncertain sources; and a considerable portion of the payments for patients has been diverted from the more legitimate object of providing for the proper treatment and comfort of those on whose account they were made, into furnishing accommodation for others. In this way, a large proportion of the public asylum accommodation in Scotland has been provided from monies levied directly on the friends of the insane, by making the payments on their account considerably exceed the expenditure; instead of by the fairer course of assessing the community. This procedure is well illustrated by the history of the Dundee Asylum. A sum, amounting to 7706*l*. 10*s*. 8*d*., having been raised by charitable contributions, the asylum was erected at a cost of 8493*l*. 9*s*. 6½*d*. Accordingly, when opened for the reception of patients in 1820, a debt had been contracted of 786*l*. 18*s*. 10½*d*. In 1859, the sum expended on land and buildings had increased to 35,262*l*. 3*s*. 2*d*., of which sum 5640*l*. 1*s*. 4½*d*. had been obtained through further charitable contributions, and 4144*l*. 8*s*. 9*d*. had been borrowed. It thus appears that during the 39 years which have elapsed since the opening of the asylum, the patients have contributed 17,771*l*. 2*s*. 4½*d*. beyond the cost of their maintenance; and this sum has been spent, not for the special benefit of these patients, but in providing accommodation for the district. In other words, a public want has been supplied from the private funds of those who, perhaps of all the community, were the least able to afford the sacrifice." (p. lii.)

The following remarks concerning the celebrated Crichton Institution, at Dumfries, also deserve to be noted, as bearing upon the same subject.

"The plan of the building of the Crichton Institution is such that many of the galleries must necessarily have a dark and gloomy appearance; nevertheless, they are capable of being rendered much more cheerful than they are. There is especially room for improvement of the furniture, in which there is a pervading bareness, both in quantity and quality, throughout the establishment. It is generally objected to private asylums that the principle of profit is allowed to interfere with the comforts of the patients; but in the present case, it appears that the necessity for providing funds for the extension of the pauper departments of the asylum is permitted to swallow up an undue share of the payments made for private patients. The apartments of the highest class of patients, though comfortably furnished in essential respects, are scarcely fitted up in accordance with the previous habits of the patients, or their position in society; but it is in the lower galleries that the want of articles necessary even for comfort is most apparent. The flagged day-rooms there have neither matting nor carpeting; the lowest rate at which patients are now admitted is 50*l.* a year." (p. lviii.)

The Commissioners report at length on the condition of the different public and private asylums in Scotland, and on criminal and alien lunatics; and they terminate their report with a few comments on the property of lunatics.

Affixed to the report are several appendices containing returns and tables showing the number and distribution of lunatics in the different asylums and poorhouses in Scotland, the cost of pauper lunatics, pecuniary allowances to single patients in private houses, &c., and the movement of patients in asylums; also the general reports of the Visiting Commissioners on the condition of single patients.

The tables which refer to the movement of patients in asylums, show the results for 1858 and 1859, and include returns of the monthly admissions, discharges, and deaths in public and private asylums and poorhouses; also of the length of residence of those who had recovered and those who had died, as well as of the causes of death. These returns, continued over a few years, will furnish a vast body of useful information.

The general reports of the Visiting Commissioners contain much matter of interest. We extract several paragraphs from these documents illustrative of one of the most important modes in which mental, moral, and physical degradation is fostered among the impoverished and lower classes of the population. The subject is one which cannot be too much dinned into the ears of the public.

Mr. Cockburn writes:—

"In the county of Banff there is also evidence of improvement in the condition of the pauper lunatics since last visitation. This is more particularly seen in the better house and sleeping accommodation, and in the increase of the money allowances. Their condition, however, still requires much amelioration. Nine patients, in particular, were found in a very unsatisfactory state, and appropriate changes have been recommended. Among these are two illegitimate idiot sisters living under charge of an imbecile mother and of an aunt who is periodically insane. This arrangement I cannot but regard as highly unsatisfactory, if not unsafe. Their dying grandmother is also in the house, the general aspect of which was squalid and bare. Another somewhat similar case, in respect of the guardian not being a suitable person, is that of the begging imbecile J. C., who lives with a thriftless sister said to drink, and whose bed and home were found in a filthy and comfortless state." (p. 194.)

Dr. Browne writes of the lunatics at large in the southern part of Ayrshire :—

"When viewed in their moral aspect, it is found that these classes [idiots, imbeciles, chronic lunatics, 260 in number—111 females, 149 males, chiefly living with relatives or strangers] include 3 individuals who nudify; 12 prostitutes, or idiots and imbeciles who have borne illegitimate children; and 11 drunkards. It is not for the reporter to attempt to determine how much of moral turpitude, and how much of mental infirmity, may enter into such diseased minds; but the obvious union of these elements produces such an amount of degradation, such an outrage upon decency, as to tax belief. There is, for example, a case among several others, where a squinting, hideous, dirty, drunken imbecile has borne three illegitimate children, all of whom were idiots, to different fathers. One of them, still lower in the intellectual scale than his parent, is in the poorhouse; another was burned to death; the fate of the third could not be ascertained. The mother is supposed still to prostitute herself, and to share the wages of her iniquity with her mother, in whose house she lives. It has been most erroneously supposed that a disposition existed to urge too stringently the seclusion of cases where neither danger nor violence was apprehended. The accusation should be reversed, and blame attached either to the Act or to the Board of Lunacy for sanctioning the continued liberty of such an individual as the one described. The limited powers of the Board may be well illustrated by the fact that this woman, undoubtedly insane, living upon charity and crime, procreating idiots worse than herself, is beyond their control from not being *at present* in receipt of parochial relief." (p. 198.)

Mr. Cockburn again writes :—

"In the parish of W., an illegitimate girl, aged eleven, a pauper, is boarded with, and is under the entire charge of her grandmother, M. T., an irascible, peculiar woman, who is on the roll as a pauper lunatic. She will not allow the child to attend school, nor at any time to go out alone. When the grandmother goes out herself, the girl is locked into the house. The girl is healthy and intelligent, but growing up uneducated, and in the society of a fatuous old woman and an aunt with an infant bastard. M. T. was in prison six years ago on suspicion of child murder, by putting another bastard grandchild under the ice." (p. 203.)

Mr. Mitchell writes :—

"The Zetlanders, as a general characteristic, are a sober and virtuous people. Nevertheless I have reported on the cases of seven fatuous mothers, who had borne illegitimate children. The child of A. F., one of these women, is an idiot; and J. M., who is herself illegitimate, has borne three bastard children; one of the three being a complete idiot, and a second one imbecile. Of the third I know nothing. Altogether I have reported on five fatuous persons, who are the illegitimate offspring of fatuous mothers, some of these last being now dead." (p. 21).

Other illustrations of the same class may be found in these reports; but we here close our analysis of the very valuable Second Annual Report of the Scotch Lunacy Commissioners.

---

### Art. VII.—POPULAR PHYSIOLOGY.—THE NERVOUS SYSTEM*

